# A Low-Frequency MEMS Magnetoelectric Antenna Based on Mechanical Resonance

**DOI:** 10.3390/mi13060864

**Published:** 2022-05-30

**Authors:** Yinan Wang, Zhibo Ma, Guanglei Fu, Jiayan Wang, Qi Xi, Yuanhang Wang, Ziqiang Jia, Guhao Zi

**Affiliations:** 1 The Ministry of Education Key Lab of Micro/Nano Systems for Aerospace, Northwestern Polytechnical University, Ministry of Education, Xi’an 710072, China; wangyinan@mail.nwpu.edu.cn (Y.W.); wangjiayan@mail.nwpu.edu.cn (J.W.); qqxiqi001@mail.nwpu.edu.cn (Q.X.); wwyyhh@mail.nwpu.edu.cn (Y.W.); jiaziqiang@mail.nwpu.edu.cn (Z.J.); 2082990303@mail.nwpu.edu.cn (G.Z.); 2 Shaan’xi Key Lab of MEMS/NEMS, Northwestern Polytechnical University, Xi’an 710072, China; 3 Training Center for Engineering Practices, Northwestern Polytechnical University, Xi’an 710072, China

**Keywords:** magnetoelectric coupling, microfabrication process, MEMS mechanical antenna

## Abstract

Antenna miniaturization technology has been a challenging problem in the field of antenna design. The demand for antenna miniaturization is even stronger because of the larger size of the antenna in the low-frequency band. In this paper, we consider MEMS magnetoelectric antennas based on mechanical resonance, which sense the magnetic fields of electromagnetic waves through the magnetoelectric (ME) effect at their mechanical resonance frequencies, giving a voltage output. A 70 μm diameter cantilever disk with SiO_2_/Cr/Au/AlN/Cr/Au/FeGaB stacked layers is prepared on a 300 μm silicon wafer using the five-masks micromachining process. The MEMS magnetoelectric antenna showed a giant ME coefficient is 2.928 kV/cm/Oe in mechanical resonance at 224.1 kHz. In addition, we demonstrate the ability of this MEMS magnetoelectric antenna to receive low-frequency signals. This MEMS magnetoelectric antenna can provide new ideas for miniaturization of low-frequency wireless communication systems. Meanwhile, it has the potential to detect weak electromagnetic field signals.

## 1. Introduction

With the progress of portable communication technology, antenna miniaturization technology has acquired more attention [1,2]. The demand for antenna miniaturization is even stronger because of the larger size of the antenna in the low-frequency band. The large antenna sizes associated with the low-frequency band restrict the development of wireless communication systems [3]. The current antenna miniaturization mechanism relies on electromagnetic resonance; hence, the antenna size is typically larger than one-tenth of the wavelength [4,5,6]. In summary, the development of new antenna miniaturization mechanisms poses a significant research direction for the antenna design field [7,8].

Mechanical antennas are a new type of antenna, which implement the coupling of the electromagnetic field and current by use of the mechanical movement of charge or magnetic dipole [9,10]. The new radiation principle makes it possible to change the physical size constraint associated with the conventional antenna wavelength; therefore, it is possible to reduce the antenna size significantly [11,12]. A magnetoelectric (ME) mechanical antenna combines the piezoelectric and magnetostrictive effects and realizes the mutual conversion of the electromagnetic field and oscillating current through the use of a distinctive magnetoelectric coupling structure. Unlike the conventional electrically small antennas that resonate through electromagnetic waves, ME antennas use magnetic dipole moment oscillations and generate mechanical resonance. This approach breaks the complementary relationship between antenna size and wavelength and, so, can lead to significantly reductions in antenna size [13].

In recent years, with the development of magnetostrictive thin films [14,15], piezoelectric thin films [16], and multiferroic heterojunction technologies [17,18], the magnetoelectric coupling effect at the micro and nano scales has received wide attention. The magnetoelectric coupling structure can realize the dynamic transformation of electric field and magnetic field energy [19,20]. For sensors [21,22,23], applications have laid the foundation for magnetoelectric mechanical antennas [24,25]. Recently, mechanical antennas based on a magneto-electric coupling effect have been demonstrated at very high frequency (VHL) and ultra-high frequency (UHL) [13,26,27]. This magnetoelectric mechanical antenna breaks the bounded relationship between wavelength and antenna size. Compared to advanced miniaturized antennas, this magnetoelectric antenna is one to two orders of magnitude smaller. Currently this miniaturized antenna based on the coupling effect is oriented to existing 5G, WIFI, or other high-frequency bands, and there is a temporary lack of systematic research on low-frequency MEMS antennas based on the magnetoelectric coupling effect.

For this paper, we fabricated a low-frequency micromechanical antenna based on mechanical resonance, which converts electromagnetic waves into oscillating currents through a magnetoelectric structure for low-frequency electromagnetic wave reception. During reception, the magnetostrictive film of the magnetoelectric antenna converts the magnetic component of the electromagnetic field into mechanical vibrations and generates an oscillating voltage in the lower piezoelectric film. This approach facilitates the reception of low-frequency electromagnetic wave signals through mechanical resonance of the micromechanical antenna, rather than by electromagnetic resonance. Therefore, the micromechanical low-frequency antenna size is smaller than the existing low-frequency antenna. This micromechanical antenna demonstrates potential, not only for miniaturized communication systems, but also for integrating different frequency antennas.

## 2. Materials and Methods

### 2.1. MEMS Magnetoelectric Antenna Concepts

In this section, we describe the fabrication of and characterize a miniaturized LF magnetoelectric coupling antenna based on mechanical resonance. The MEMS magnetoelectric antenna consists of one layer of aluminum nitride (piezoelectric material) and one layer of FeGaB (magnetostrictive material); it is based on the magnetoelectric coupling effect, which converts electromagnetic waves into a dynamic voltage signal.

**Figure 1:**
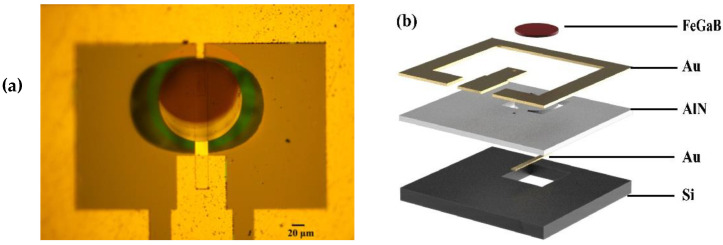
(**a**) Optical microscope photograph. (**b**) The structure and layers of the of the MEMS magnetoelectric antenna.

The magnetic component of an electromagnetic wave can be detected by the magnetostriction layer, inducing mechanical vibrations through the magnetic coupling effect. When the mechanical vibrations transfer to the piezoelectric thin-film, due to the piezoelectric effects, a dynamic voltage signal is generated. Based on the above principles, using the five-masks micromachining process, a MEMS magnetoelectric antenna with a SiO_2_/Cr/Au/AlN/Cr/Au/FeGaB stack was fabricated on a 300 μm double-side-polished silicon (1,0,0) chip. A photograph of the MEMS magnetoelectric is shown in Figure 1a.

When the magnetoelectric-coupled MEMS magnetoelectric antenna operates, the magnetostrictive material (FeGaB) senses the magnetic component in the external electromagnetic field and converts it into stress–strain in the piezoelectric film (AlN). This process generates a mechanical vibration and converts the stress–strain into an oscillating voltage signal, thus converting the electromagnetic field into an oscillating voltage. The dimensions and structure of the MEMS magnetoelectric antenna are shown in Figure 1b.

### 2.2. MEMS Magnetoelectric Antenna Sample Fabrication

**Figure 2:**
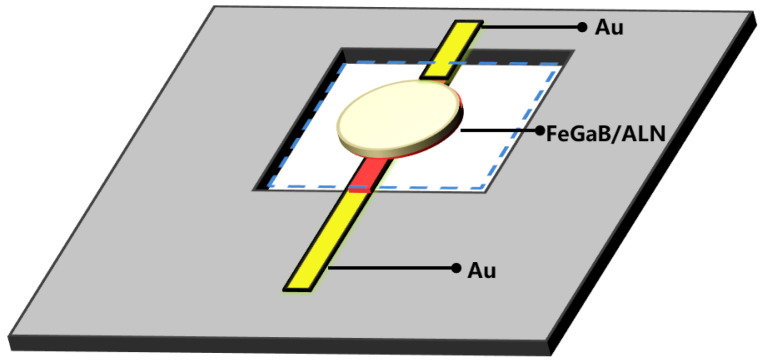
The structural dimensions of the developed MEMS magnetoelectric antenna.

Using the five-masks micromachining process, a MEMS magnetoelectric antenna with a SiO_2_/Cr/Au/AlN/Cr/Au/FeGaB stack was fabricated on a 300 μm double-side-polished silicon (1,0,0) chip. A photograph of a MEMS magnetoelectric antenna is shown in Figure 1. A SiO_2_ layer was prepared by thermal oxidation of the silicon wafers, following which the gold film was sputter-deposited and patterned using the lift-off process to the lower electrode of the MEMS magnetoelectric antenna. Next, c-axis AlN films were prepared by magnetron sputtering, after performing lithography, and the vias were etched using high-temperature phosphoric acid to connect the bottom electrode. Thereafter, the gold film was evaporated and plated, instead of being sputtered. The upper structural electrode was formed using the lift-off process. Then, the silicon dioxide on the lower surface was removed by hydrofluoric acid. The magnetostrictive material, FeGaB, was then sputtered. The patterning was completed through the lift-off process. Finally, the structure was released by deep silicon etching on the back side. The structural dimensions of the MEMS magnetoelectric antenna are shown in Figure 2.

In detail, the MEMS magnetoelectric antenna construction process is as follows. First, a double-side-polished silicon wafer (100) with a thickness of 300 μm is taken; after standard cleaning, a silicon dioxide layer of 100 nm was grown on the surface using a thermal oxidation process. The silicon dioxide layer acts as an insulating and buffer layer. Gold was selected as the lower electrode and support structure for the lithography process, followed by sputtering of Cr (10 nm) and gold film (100 nm), then ultrasonic stripping in an acetone solution. The length of the overhanging part of the gold beam was 100 μm, its width was 10 μm, and its thickness was 100 nm.

Next, AlN films were sputtered and deposited with the following process parameters: 0.25 Pa pressure, 20 sccm Ar flow rate, 10 sccm N_2_ flow rate, and 350 W RF power. This ensured AlN film growth along the c-axis. The wafer was cleaned, lithographed, and the ALN vias were etched by high-temperature (80 °C) phosphoric acid to connect the bottom electrodes. The piezoelectric film (AlN) was 500 nm thick. The underlying gold film was separated by an insulating AlN film, and the lower gold electrode was connected to the external pads through a window reserved during AlN patterning when preparing the upper gold electrode. The gold film was prepared using the vacuum vapor deposition method, ensuring that the upper and lower electrodes were connected. The thickness of the gold film was 100 nm and the adhesion layer was 10 nm Cr. The length of the overhanging part of the gold beam was 30 μm, its width was 8 μm, and its thickness was 100 nm. After that, the back-side silicon oxide was removed using hydrofluoric acid. The silicon wafer was cleaned and subjected to a photolithography process.

Then, the sputtering of magnetostrictive thin-film FeGaB was followed by an ultrasonic stripping process in acetone solution. The Fe_7_Ga_2_B_1_ sputtering process parameters were as follows: 0.3 Pa pressure, 20 sccm Ar flow, and 50 W RF power. After sputtering, the magnetostrictive layer was annealed at a temperature of 200 °C, with a bias magnetic field of 10 mT. The FeGaB film was a 70 μm diameter resonant disc. The silicon wafer was cleaned, and we performed the photolithography process. Next, we performed back cavity etching to release the MEMS resonant structure. The etching rate was reduced in the final stage of etching to protect the structure.

**Figure 3:**
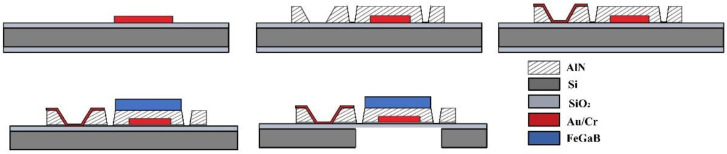
Machining process flow for the developed MEMS magnetoelectric antenna.

Finally, the passivation process was performed, and scribing occurred after cleaning the silicon wafer. The magnetoelectric heterostructure resonant disc structure had a diameter of 70 μm and a thickness of 1 μm (AlN 500 nm FeGaB 500 nm), while the lower gold electrodes had a length of 150 μm and a thickness of 100 nm. The specific machining process for the MEMS magnetoelectric antenna is detailed in Figure 3. The thicknesses and Young’s moduli of the various materials used are listed in Table 1.

### 2.3. Measurement Setup

To accurately evaluate the performance of the magnetoelectric response of the MEMS magnetoelectric antenna in a weak magnetic environment, we established a dedicated test environment for magnetic shielding.

First, a customized high-precision magnetic shielding device was used to provide a weak magnetic environment for device testing. The high-precision magnetic shielding device includes a sealed bottom at one end and a live cover at the other end. The inner and outer layers of the high-precision magnetic shielding device are aluminum, and the middle layer is a high-permeability permalloy. The device must be kept magnetically clean. Before testing, the magnetic shielding barrel needed to be demagnetized. To eliminate self-induced interference in the wire, the wire current input to the Helmholtz coil needs to be provided by a voltage-to-current conversion circuit to eliminate the weak magnetic field generated by the current in the wire from affecting the accuracy of the test results. To further exclude external magnetic interference and accidental errors, a vacant interface was used at the test port as a control group to verify the validity of the received signal from the MEMS magnetoelectric antenna.

Under the weak magnetic field, the electrical signal output from the MEMS magnetoelectric antenna was very weak, and an NI data acquisition card was used to collect the output voltage information, and the data were analyzed and processed using LabVIEW 2018 software (National Instruments, Austin, TX, USA). The magnetic shield barrel, Helmholtz coil, high-precision power supply (Tektronix 6211), and test circuit formed the hardware part of the MEMS magnetoelectric antenna magnetoelectric response test system.

**Figure 4:**
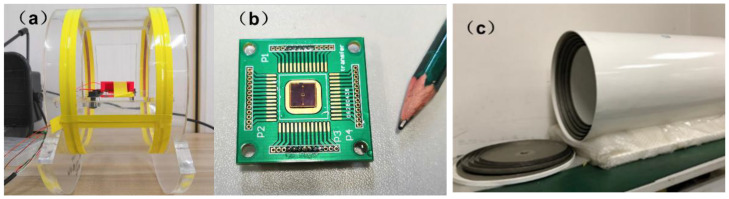
Composition of the test device: (**a**) Helmholtz coil; (**b**) MEMS magnetoelectric antenna; and (**c**) electromagnetic shielding cylinder.

**Figure 5:**
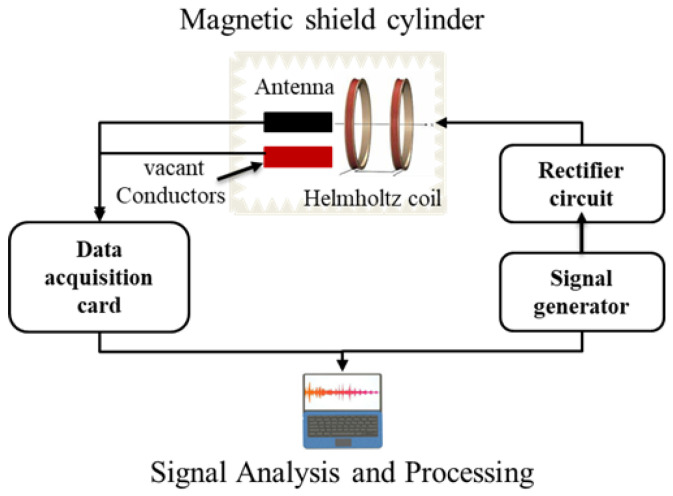
Weak magnetic test system principle.

The sampling rate of LabVIEW 2018 software was 200,000, where each sampling point was tested with 30 data, and the weighted average was taken as the test result. The spectrum graph of the channel displayed the test results of the device output and, to exclude the error of the wire itself, channel two was the vacant wire output signal. When the channel one and two signals were equal, the test result was the weak magnetic error generated by the wire itself, which was not included in the data. The set-up and principle of the test system are shown in Figure 4 and Figure 5, respectively.

Main steps of the testing process as follows:

(1) Place the MEMS magnetoelectric antenna chip in the magnetic constant position of the Helmholtz coil after cleaning, with the electromagnetic field vertical to the MEMS magnetoelectric antenna.
(2) Gradually decrease the current strength of the input Helmholtz coil in suitable steps, and record and analyze the output signal.
(3) Collect the vacant wire data of channel 2, compare with the test data, and exclude the wire and chance errors.

## 3. Results

### 3.1. Signal Analysis and Processing

The output signal of the LabVIEW 2018 was compared with that of channel 2 and then collated to obtain the output voltage signal under different magnetic field frequency magnitudes. The value of the output through LabVIEW was given in dB, and its calculation formula is:

(1) $$dB=20\mathrm{lg}\frac{I_{2}}{I_{1}},$$

where $I_{1}$ is the reference voltage and $I_{2}$ is the output voltage.

The MEMS magnetoelectric antenna output electrical signal is:

(2) $$U=10^{\left(\frac{dB}{20}\right)}.$$

In addition, as the input current signal is sinusoidal, the peak voltage *U*_1_ in the test result is:

(3) $$U_{1}=\sqrt{2}U.$$

The sensitivity of the MEMS magnetoelectric antenna to the magnetic signal is:

(4) $$Z=\frac{n}{1000U_{1}},$$

where *n* is the size of the alternating magnetic field.

**Figure 6:**
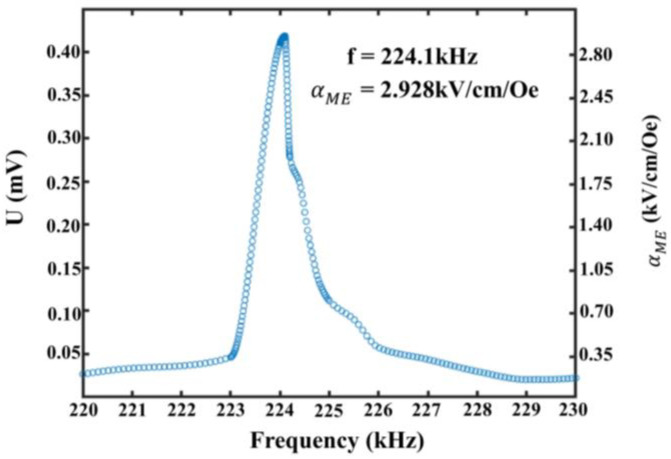
MEMS magnetoelectric antenna output signal and ME coefficient.

The magnetoelectric coupling (ME) voltage output was sensed by the NI data acquisition card under H_RF_ excitation of approximately 286.11 nT (Wb/m^2^), with the electromagnetic field vertical to the MEMS magnetoelectric antenna, as shown in Figure 6.

The magnetoelectric coupling coefficient $\alpha_{ME}$ is calculated as:

(5) $$\alpha_{ME}=\frac{\alpha V}{\alpha_{RF}\times T},$$

where *T* is the thickness of AlN, $\alpha_{RF}$ is the Ac magnetic field, and the ME coefficient is $\alpha_{ME}$.

The MEMS magnetoelectric antenna magnetoelectric coupling coefficient was 2.928 kV/cm/Oe. It is worth noting that the magnetoelectric coupling coefficients shown in Figure 6 were obtained without any bias magnetic field, which is comparable to the recently reported results tested at optimal bias magnetic fields. [14,23,30].

The MEMS magnetoelectric antenna a resonant frequency can be expressed as:

(6) $$f\propto \frac{1}{w}\sqrt{\frac{\Sigma E_{n}}{\Sigma \rho }},$$

where *f* is the working frequency of the MEMS magnetoelectric antenna, *W* is the width of the resonator, *E_n_* is the equivalent Young’s modulus, and Σρ is the equivalent density MEMS magnetoelectric antenna. By adjusting the resonant structure, it is possible to realize the fabrication of a MEMS magnetoelectric antenna with different frequencies on the same chip. As can be seen from Figure 5, the magnetoelectric coupling coefficient was 2.928 KV/cm/Oe at a resonant frequency of 224.1 kHz. Meanwhile, a clear peak (0.4188 mV) was observed, which demonstrates that the proposed MEMS magnetoelectric antenna based on the magnetoelectric coupling effect can achieve LF electromagnetic wave reception.

### 3.2. Sensitivity and Detection Limit

**Figure 7:**
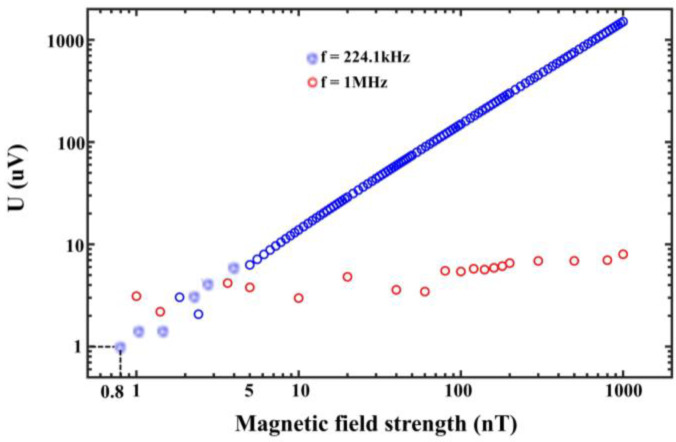
MEMS magnetoelectric antenna output voltage signal versus external magnetic field.

The induction limit of the MEMS magnetoelectric antenna to the magnetic field was characterized in the absence of a bias magnetic field, and the results are shown in Figure 7.

As shown in Figure 7, the voltage signal generated by the MEMS magnetoelectric antenna without a bias magnetic field is characterized with the maximum output signal strength at the mechanical resonant frequency (224.1 kHz). The output magnetic field strength of the test system is 0.8 nT–1000 nT, and the magnetic field strength and output voltage signal strength are shown in the blue curve. The output signal under alternating magnetic field excitation at 1 MHz is shown in the red curve, and the output signal strength is about 1–8 μV, so the MEMS magnetoelectric antenna shows no sensitivity to magnetic field excitation at 1 MHz. In addition, at 0.8 nT (800 pT), the output signal strength of the MEMS magnetoelectric antenna is about 1 µV. The MEMS magnetoelectric antenna detection limit is about 800 pT with a limit detection voltage of 1 μV. It is noteworthy that there is still signal output by reducing the magnetic field strength, but the output signal is drowned in the circuit noise. Therefore, increasing the output signal strength of the magneto-electric coupling structure enables the MEMS magnetoelectric antenna to receive fainter electromagnetic signals.

In summary, the MEMS magnetoelectric antenna works in mechanical resonant frequency and realization of low-frequency specific signals (224.1 kHz) electromagnetic field signal reception. It can also be seen that this MEMS magnetoelectric antenna has the property of being sensitive to specific frequency electromagnetic waves and the output signal strength varies linearly with the excitation magnetic field, thus having the potential to become an ultra-high-sensitivity magnetic sensor.

## 4. Conclusions

In this paper, we fabricated a MEMS magnetoelectric antenna based on mechanical resonance, which converts electromagnetic waves to oscillating currents through a magneto-electric coupling effect for low-frequency electromagnetic wave reception. A 70 μm diameter cantilever disk with SiO_2_/Cr/Au/AlN/Cr/Au/FeGaB stacked layers is prepared on a 300 μm silicon wafer using the five-masks micromachining process. We characterize the magnetoelectric coupling coefficient, resonant frequency, and sensitivity of the MEMS magnetoelectric antenna to electromagnetic field excitation at different frequencies. The magnetoelectric coupling coefficient was 2.928 kV/cm/Oe at the mechanical resonant frequency of 224.1 kHz, while the detection limit is 800 pT at 1 µV. We found that the magnetoelectric antenna is only sensitive to electromagnetic waves at frequencies near its own mechanical resonance frequency, and the detection limit of the portable system can be significantly improved by an array structure or vacuum encapsulation. In addition, this MEMS magnetoelectric antenna has the property of being sensitive to specific frequency electromagnetic waves and the output signal strength varies linearly with the excitation magnetic field, thus having the potential to become an ultra-high-sensitivity magnetic sensor.

## Figures and Tables

**Table 1:** List of used materials thicknesses and Young’s moduli.

Material	AlN	FeGaB	Cr	Au	SiO_2_
Thicknesses (nm)	500	500	10	100	100
Young’s modulus (GPa)	400	55	140	78.9	70
Density (g/cm^3^)	3.3	7.86	7.19	19.28	2.20
References	[13]	[13]	[28]	[29]	[28]
